# Social Media Use in Adolescents: Bans, Benefits, and Emotion Regulation Behaviors

**DOI:** 10.2196/64626

**Published:** 2024-11-04

**Authors:** Kelsey L McAlister, Clare C Beatty, Jacqueline E Smith-Caswell, Jacqlyn L Yourell, Jennifer L Huberty

**Affiliations:** 1 Fit Minded, Inc, Phoenix, AZ, United States, 1 6029356986

**Keywords:** adolescent social media, social media bans, emotion regulation, youth, adolescent, media use, social platform, social network, self-regulation, behavioral health, mental health, digital health, technology, digital literacy

## Abstract

Social media is an integral part of adolescents’ daily lives, but the significant time they invest in social media has raised concerns about the effect on their mental health. Bans and severe restrictions on social media use are quickly emerging as an attempt to regulate social media use; however, evidence supporting their effectiveness is limited. Adolescents experience several benefits from social media, including increased social connection, reduced loneliness, and a safe space for marginalized groups (eg, LGBTQ+) to interact. Rather than enforcing bans and severe restrictions, emotion regulation should be leveraged to help adolescents navigate the digital social environment. This viewpoint paper proposes a nuanced approach toward regulating adolescent social media use by (1) discontinuing the use of ineffective bans, (2) recognizing the benefits social media use can have, and (3) fostering emotion regulation skills in adolescents to encourage the development of self-regulation.

## Introduction

Social media, defined as technologically based platforms using public, continually accessible, algorithmic-based social interactions, is an integral part of adolescents’ daily lives [1]. Ninety percent of adolescents (aged 13‐19 years) in the United States report that they have a social media platform [2], with more than half using these platforms for more than 4 hours daily [3]. The significant time adolescents invest in social media has raised concerns about the impact on their mental health and well-being, prompting various local and national initiatives advocating for bans and severe restrictions on social media usage. Yet, research suggests that time on social media is not predictive of mental health outcomes and bans often fail to achieve their intended purpose, with little to no improvement in mental health or academic performance [4,5]. Evidence across various types of bans for adolescents (eg, smartphone bans, banning transgender youth from sports participation, and book bans) indicates that such restrictions carry several negative consequences for their mental health [6-9]. In this viewpoint paper, we advocate that instead of enforcing bans and severe restrictions, we focus our efforts on fostering emotion regulation in adolescents, so they develop skills specifically tailored for self-regulating their social media interactions. This redirection is crucial given the low efficacy of bans, the numerous benefits of social media use, and the necessity of developing emotion regulation skills for healthy development.

## Limitations of Bans and Severe Restrictions

Social media bans and severe restrictions are a rigid, ineffective response to evolving issues that warrant continuous evaluation. Social media bans inhibit adolescent psychosocial needs by keeping adolescents from a source of meaningful connection without offering a valuable alternative. Strict social media restrictions and bans for adolescents have resulted in several negative consequences, such as instilling feelings of isolation, fostering rebellion against authority, and contributing to underdeveloped digital literacy skills [10]. Additionally, legislators have been attempting to impose time restrictions on social media use for minors [11,12] but have faced challenges in passing such regulations due to constitutional concerns. Even if implemented, these time restrictions do not address the quality of adolescents’ social media interactions and other contextual factors, which are critical for evaluating their impact on mental health. The current evidence on the effectiveness of bans in improving adolescent mental health remains weak and inconclusive [13-15]. A recent scoping review found that only 6 of the 22 studies examined the effects of mobile phone bans on mental health and well-being in schools [5]. Of these, 2 included anecdotal support for banning mobile phones while 4 found no evidence to support bans for adolescent mental health and well-being [5]. Further, studies on other adolescent outcomes (eg, academic performance) were mixed, likely due to variation in the type of ban (eg, partial, full, and social media only) and the aspect of social media (eg, time spent, highly visual apps, and instant messaging–based apps) investigated, which hinders definitive conclusions [16]. Future work examining specific types of restrictions and bans is needed to elucidate their efficacy. Taken together, the limited evidence supporting bans underscores that bans are not the most effective solution for improving adolescent well-being.

In spite of the limited empirical support, social media bans and severe restrictions continue to emerge in an attempt to ameliorate adolescent mental health concerns. Recent examples of such initiatives include a Florida law banning social media use for those aged 14 years and younger, and requiring parental consent for 12-15 years old [11]. Similarly, the Los Angeles Unified School District, the second largest in the United States (with more than 429,000 students), has banned cellphone and social media use during school hours for K-12 students [17]. This trend extends beyond the United States, as France proposed a law to ban social media use for those aged 15 years and younger [18]. Adding to these measures, US Surgeon General Dr Vivek H Murthy called on Congress to implement warning labels on social media platforms, similar to those found on cigarette packages. Dr Murthy argued for swift action, citing social media as “an important contributor” to the mental health crisis among young people. This characterization oversimplifies social media use and neglects the diverse experiences adolescents can have on social media, including positive ones.

## Benefits of Social Media

Social media is often regarded as solely negative for adolescents, overlooking its many benefits. For example, a thematic meta-synthesis of *qualitative* studies demonstrated that social media had a nuanced impact on adolescent well-being, revealing both positive effects (eg, promoting learning) and negative effects (eg, impacting mood through exposure to upsetting content) [19]. Social media fulfills several psychological and social needs for adolescents, such as self-expression, social validation, and peer interaction [20-22]. A survey conducted by EdWeek Research Center among 1054 high school adolescents found that one-third of adolescents report feeling less alone because of social media and 72% report that social media has either no impact or a positive impact on their mental health [23]. Adolescents value social media for its unique benefits, such as overcoming shyness, building relationships, staying connected over distances, and facilitating group interactions and shared experiences that in-person communication cannot always provide [19]. Evidence also shows that social media can provide a safe space for specific adolescent populations, such as those in the LGBTQ+ community [24]. For these marginalized groups, dedicated web-based communities serve as crucial support systems [25]. Additionally, social media can connect adolescents with varied interests, such as fandom communities where adolescents can engage with others who have shared interests in books, films, or others [26]. In a recent study of fandom communities, sexual and gender minority youth reported that web-based communities contribute to their identity development by providing a safe and anonymous space, as well as offering validation and normalization [26]. While social media may foster various types of connections, most existing naturalistic research to date has focused on negative mental health outcomes. Consequently, there is a dearth of research examining its potential positive effects.

Social media platforms can be leveraged to deliver interventions providing mental health support for adolescents. Various interventions show promise, from brief, targeted approaches [27] to ongoing peer support networks [28]. For example, single-session interventions integrated into popular social media platforms demonstrate potential in addressing acute mental health needs, such as self-harm [29]. Exposure to personal recovery narratives on social platforms can reduce suicidal ideation [30]. Furthermore, the power of peer support on these platforms appears to be mutually beneficial, as providing support to others online can lead to improvements in one’s own mental health and coping strategies [31,32]. These findings emphasize the benefits of social media and underscore the need for a more nuanced approach to adolescent use. Greater emphasis should be placed on fostering positive experiences online and mitigating negative ones. Adolescents need assistance in developing the skills necessary to navigate the complex landscape of social media, including the ability to understand and manage their emotions.

## Emotion Regulation in Adolescence

Adolescence is marked by significant psychosocial shifts, including the development of identity and independence away from parents and caregivers [33]. These shifts are accompanied by various emotional challenges (eg, increased social and academic pressures). Understanding and effectively managing emotions is critical for navigating social media experiences in a healthy manner. This skill set, known as emotion regulation, is broadly defined as one’s ability to intentionally or unintentionally influence or manage one’s emotions [34]. The process model of emotion regulation, developed by Gross, provides a framework for understanding how individuals regulate their emotions [35,36]. This model identifies 5 stages in the emotion regulation process: situation selection, situation modification, attentional deployment, cognitive change, and response modulation [35]. Each stage represents a point at which individuals can intervene to influence their emotional experiences.

*Situation selection* involves choosing to approach or avoid situations that are likely to elicit emotional responses [35,36]. Situation selection can precede the onset of an emotion, meaning people can elect into or out of situations that elicit emotional responses. This is unique to the other stages of emotion regulation which occur after the emotion has already been elicited. By identifying and avoiding situations (or social media content) that may bring about negative emotional experiences, one can prevent those negative emotions from arising. For example, an adolescent who feels anxious might avoid engaging with highly visual and stimulating apps (eg, TikTok) to prevent exacerbating their anxiety, thereby proactively managing their emotional state (ie, situation selection). Alternatively, the adolescent might select social media platforms that induce positive emotional experiences (eg, a mindfulness-based app or a communication platform). Depending upon the developmental stage of the adolescent, identification and situation selection could either be self-directed or could be taught and supported by parents or caregivers.

Several emotion regulation strategies can be deployed once an emotion has been triggered. *Situation modification* and *attentional deployment* are 2 strategies that leverage behavior once an emotion has been elicited, either by redirecting attention or manipulating a situation to better suit one’s needs [35,36]. To do so, adolescents must develop the ability to recognize their current emotional experiences. *Situation modification* refers to altering aspects of the situation to minimize negative emotional experiences or foster positive ones. In the context of social media use, this could include recognizing negative emotional shifts when they occur while using social media (as generative content means less direct control over what is viewed) and navigating away from it (eg, leaving the app or website). For example, TikTok, a popular social media app, contains categories of content for users to engage with. Adolescents could modify their emotional experience while staying on the app by searching strategically for content that elicits the desired emotion. *Attentional deployment* includes directing one’s attention toward or away from emotionally distressing scenarios and could include focusing on positive aspects, such as comments that foster connectedness or encouraging aspects of a platform.

*Cognitive change* refers to reappraising one’s attitudes or beliefs toward a situation, which then alters the emotional value and importance [35,36]. In the context of social media, *cognitive change* could include caregiver and adolescent discussions about the role and value of social media in one’s life. The examination of those beliefs can then foster the ability to challenge or change previously unexamined assumptions about social media use. This could be an opportunity to reframe or correct held beliefs on social media, especially during adolescence where reinforcement from peers is developing and particularly sensitive. For example, if an adolescent feels pressure to post about their achievements to gain validation from their peers, they may feel distressed if they have low engagement on their posts. Caregivers can help by discussing how self-worth is not tied to online validation and encouraging activities that build self-esteem.

Lastly, *response modulation* includes adjusting one’s behavioral response to an emotion, either by inhibiting or amplifying it [35,36]. Given the interactive nature of social media, this modulation is particularly relevant for content creation and engagement. In this context, response modulation includes an initial emotion to content (internal) that is then either inhibited or amplified through a behavioral response (external). For example, an adolescent who feels excited after receiving a compliment on their post might amplify this positive emotion by engaging more with the content (ie, liking and commenting on other posts), sharing their excitement with friends, or posting more frequently to maintain positive interaction. Conversely, if the adolescent receives a negative comment that makes them feel upset, they might inhibit their emotional response by choosing not to respond to the comment or by taking a break from social media to calm down. By modulating their responses, users can shape their social media experience to be more positive and constructive.

The application of Gross and colleagues’ process model to emotion regulation presents an opportunity for adaptive social media use behaviors more so than bans. These suggestions frame one aspect of the social media use experience. An additional aspect to consider is the influence of user-driven content on algorithm-based applications. A study found that, although adolescents might understand that their actions influence the algorithm, thus determining the content they see, they often do not adjust their social media behaviors or clearly express their understanding of how algorithms work [37]. To effectively support emotion regulation in this context, it is crucial to help adolescents bridge the gap between their awareness of algorithmic influences and practical strategies for managing their social media use.

Another consideration is the role that caregivers play in helping adolescents develop and use effective emotion regulation strategies. Caregivers can aid in managing social media use and emotion regulation even while adolescents develop autonomy and become less reliant on the caregiver’s guidance throughout the adolescent years. For example, research shows that parents use social networking sites to maintain communication with their adolescents [38], highlighting how social media serves as a key avenue for fostering dialogue and connection during this developmental phase. Regulation skills can be enhanced both indirectly through modeling [39] and directly through active caregiver involvement and discussions about media content [40], which can then foster critical thinking and self-regulation in adolescents [41].

## Suggested Call to Action

Taken together, social media is an integral part of modern adolescent life. We encourage the following 4 calls to action as a summary of this viewpoint:

- *Continue research into the specific and differential effects of social media platforms and algorithms*. Social media platforms and usage patterns influence adolescent mental health outcomes differently, but regulations often overlook these distinctions. More research is needed to identify how various platforms, usage patterns, and algorithms specifically impact adolescent mental health.
- *Discontinue ineffective (and potentially harmful) social media bans*. Policy makers should explore alternative approaches to address adolescent mental health concerns.
- *Recognize social media benefits for adolescents*. Acknowledge the potential positive impacts on adolescent mental health, especially for vulnerable and minoritized groups. A balanced perspective will foster opportunities for healthy usage.
- *Promote emotion regulation strategies on social media*. Encourage the development of skills that enable adolescents to navigate digital spaces and foster positive social media experiences. The process model of emotion regulation provides an organized way to apply emotion regulation specifically to social media use behaviors. Parents, educators, and app developers should create environments and tools that help adolescents practice and strengthen their emotion regulation skills in the context of social media use.

## Conclusion

Shifts in psychosocial development throughout adolescence and navigating the social media landscape present unique challenges for adolescents, all of which can significantly impact adolescents’ emotional well-being. Yet, social media bans and severe restrictions that rely on external regulation offer little improvement in addressing these unique challenges. Further, social media bans and severe restrictions neglect the positive experiences that promote social connectedness and improve mental well-being among adolescents. Emotion regulation, which can be self-directed, offers a way forward to address these challenges while maintaining the autonomy and interests of adolescents. Parental or caregiver guidance helps adolescents become discerning consumers of media, better equipped to handle the emotional challenges posed by digital interactions. The process model of emotion regulation can serve as an initial guide to orient the identification and usage of emotion regulation skills within specific social media use behaviors. Taken together, emotion regulation in social media use offers a promising avenue forward that maintains adolescent agency while addressing growing concerns regarding adolescent mental health.
